# Adherence at 2 years with distribution of essential medicines at no charge: The CLEAN Meds randomized clinical trial

**DOI:** 10.1371/journal.pmed.1003590

**Published:** 2021-05-21

**Authors:** Nav Persaud, Michael Bedard, Andrew Boozary, Richard H. Glazier, Tara Gomes, Stephen W. Hwang, Peter Juni, Michael R. Law, Muhammad Mamdani, Braden Manns, Danielle Martin, Steven G. Morgan, Paul Oh, Andrew D. Pinto, Baiju R. Shah, Frank Sullivan, Norman Umali, Kevin E. Thorpe, Karen Tu, Andreas Laupacis, for the Carefully seLected and Easily Accessible at No Charge Medications (CLEAN Meds) study team

**Affiliations:** 1 Department of Family and Community Medicine, University of Toronto, Toronto, Ontario, Canada; 2 Li Ka Shing Knowledge Institute, St. Michael’s Hospital, Unity Health Toronto, Toronto, Ontario, Canada; 3 Department of Family and Community Medicine, St Michael’s Hospital, Toronto, Ontario, Canada; 4 Institute of Health Policy, Management, and Evaluation, University of Toronto, Toronto, Ontario, Canada; 5 Department of Family Medicine, Northern Ontario School of Medicine, Sudbury, Ontario, Canada; 6 Department of Health Policy and Management, Harvard School of Public Health, Boston, Massachusetts, United States of America; 7 Dalla Lana School of Public Health, University of Toronto, Toronto, Ontario, Canada; 8 Institute for Clinical Evaluative Sciences, Toronto, Ontario, Canada; 9 Leslie Dan Faculty of Pharmacy, University of Toronto, Toronto, Ontario, Canada; 10 Applied Health Research Centre, St. Michael’s Hospital, Toronto, Ontario, Canada; 11 Department of Medicine, University of Toronto, Toronto, Ontario, Canada; 12 Centre for Health Services and Policy Research, School of Population and Public Health, The University of British Columbia, Vancouver, British Columbia, Canada; 13 Centre for Healthcare Analytics Research and Training at St Michael’s Hospital and Vector Institute, Toronto, Ontario, Canada; 14 Department of Community Health Sciences, Cumming School of Medicine, University of Calgary, Calgary, Alberta, Canada; 15 Department of Medicine, Cumming School of Medicine, University of Calgary, Calgary, Alberta, Canada; 16 O’Brien Institute for Public Health, Cumming School of Medicine, University of Calgary, Calgary, Alberta, Canada; 17 Libin Cardiovascular Institute, Cumming School of Medicine, University of Calgary, Calgary, Alberta, Canada; 18 Women’s College Hospital Institute for Health Systems Solutions and Virtual Care, Women’s College Hospital, Toronto, Ontario, Canada; 19 School of Population and Public Health, University of British Columbia, Vancouver, British Columbia, Canada; 20 Toronto Rehabilitation Institute, University Health Network, Toronto, Ontario, Canada; 21 Department of Research and Innovation, North York General Hospital, Toronto, Ontario, Canada; 22 Division of Population and Behavioral Science, University of St Andrews, Scotland; Stanford University, UNITED STATES

## Abstract

**Background:**

Adherence to medicines is low for a variety of reasons, including the cost borne by patients. Some jurisdictions publicly fund medicines for the general population, but many jurisdictions do not, and such policies are contentious. To our knowledge, no trials studying free access to a wide range of medicines have been conducted.

**Methods and findings:**

We randomly assigned 786 primary care patients who reported not taking medicines due to cost between June 1, 2016 and April 28, 2017 to either free distribution of essential medicines (*n* = 395) or to usual medicine access (*n* = 391). The trial was conducted in Ontario, Canada, where hospital care and physician services are publicly funded for the general population but medicines are not. The trial population was mostly female (56%), younger than 65 years (83%), white (66%), and had a low income from wages as the primary source (56%). The primary outcome was medicine adherence after 2 years. Secondary outcomes included control of diabetes, blood pressure, and low-density lipoprotein (LDL) cholesterol in patients taking relevant treatments and healthcare costs over 2 years. Adherence to all appropriate prescribed medicines was 38.7% in the free distribution group and 28.6% in the usual access group after 2 years (absolute difference 10.1%; 95% confidence interval (CI) 3.3 to 16.9, *p* = 0.004). There were no statistically significant differences in control of diabetes (hemoglobin A1c 0.27; 95% CI −0.25 to 0.79, *p* = 0.302), systolic blood pressure (−3.9; 95% CI −9.9 to 2.2, *p* = 0.210), or LDL cholesterol (0.26; 95% CI −0.08 to 0.60, *p* = 0.130) based on available data. Total healthcare costs over 2 years were lower with free distribution (difference in median CAN$1,117; 95% CI CAN$445 to CAN$1,778, *p* = 0.006). In the free distribution group, 51 participants experienced a serious adverse event, while 68 participants in the usual access group experienced a serious adverse event (*p* = 0.091). Participants were not blinded, and some outcomes depended on participant reports.

**Conclusions:**

In this study, we observed that free distribution of essential medicines to patients with cost-related nonadherence substantially increased adherence, did not affect surrogate health outcomes, and reduced total healthcare costs over 2 years.

**Trial registration:**

ClinicalTrials.gov NCT02744963.

## Author summary

**Why was this study done?:**

- Medicine nonadherence is common globally, and costs borne by patients is a major barrier.
- More than 100 countries have an essential medicines list to help meet the priority health needs of the population.
- Publicly funding essential medicines is contentious in part due to the potential cost to governments.

**What did the researchers do and find?:**

- We conducted a randomized controlled trial of free essential medicines distribution in 786 people who reported trouble affording medicines in a setting where healthcare services are publicly funded.
- Free medicine distribution improved adherence to medicines appropriately prescribed after 2 years (absolute risk difference, 10.1%; 95% confidence interval (CI) 3.3 to 16.9, *p* = 0.004).
- Free distribution had inconsistent effects on surrogate health outcomes over 2 years and lowered total healthcare costs by a median of CAN$1,117 (95% CI CAN$445 to CAN$1,778, *p* = 0.006).

**What do these findings mean?:**

- Distributing a comprehensive set of essential medicines at no charge to primary care patients improved medicine adherence and reduce healthcare costs.
- These findings could help inform policy changes in countries planning to implement universal healthcare that includes access to medicines.

## Introduction

Half of prescribed medicines are not taken as directed, and low rates of adherence are seen in both low- and high-income countries, while adherence is higher in some jurisdictions with public drug programs [1,2]. Proven treatments for chronic diseases must be taken for years in order to confer a substantial benefit, but adherence is difficult to improve, especially in the long term [3]. Few interventions are effective at improving adherence [3] even in the short term, and others such as conversations with pharmacists have a waning effect [4]. The cost borne by patients is one important barrier to medicine adherence for treatments, including those that prevent cardiovascular disease and complications of HIV-AIDS [5,6]. Global calls for universal health coverage emphasize the importance of medicine access [7].

Two prior trials of eliminating out-of-pocket costs for medicines showed mixed results after 1 year. The Post-Myocardial Infarction Free Rx Event and Economic Evaluation (MI FREEE) trial of secondary cardiovascular disease prevention found a slight increase in adherence (5.4% absolute increase) and variable effects on health outcomes that tended to favor eliminating co-payments among working-age Americans with private insurance [8]. The Affordability and Real-world Antiplatelet Treatment Effectiveness After Myocardial Infarction Study (ARTEMIS) trial found that reducing co-payments for antiplatelet agents after myocardial infarction improved adherence slightly (3.3% absolute increase) and had no effect on health outcomes for Americans with commercial or government health insurance [9].

Canada is an ideal place to isolate the effect of eliminating out-of-pocket patient costs for medicines because healthcare services are generally publicly funded without co-payments, while medicine access depends on income, age, and social support status. The Carefully seLected and Easily Accessible at No Charge Medications (CLEAN Meds) trial measures the effect of free distribution of a comprehensive set of essential medicines to primary care patients in Ontario, Canada who reported cost-related nonadherence. Preliminary 1-year results indicated improved adherence (11.6% absolute increase), as well as improvements in some but not all surrogate health outcomes [10]. Here, we report the final, 2-year results of the CLEAN Meds trial to determine if ongoing free distribution of proven treatments improves long-term adherence. These final results include healthcare costs based on routinely collected health administrative data.

## Methods

### Study design

This trial was a multicenter, open-label, parallel 2-arm, superiority, individually randomized controlled trial with 1:1 allocation that was conducted at 9 primary care sites in Ontario, Canada and approved by St. Michael’s Research Ethics Board, the Huron Shores Family Health Team Research Ethics Committee, and the Laurentian University Research Ethics Board [10]. The trial was registered (NCT02744963), and a detailed protocol was published [11]. After trial initiation, the trial was extended from 1 to 2 years when additional funding became available; the first year results were published, and after the first year, we stopped using electronic bottle cap devices to measure adherence as participants in both groups did not return the devices for data collection [10]. A Data and Safety Monitoring Board reviewed serious adverse events and medication incidents to ensure that the intervention was not harming participants. The study is reported as per the CONsolidated Standards of Reporting Trials (CONSORT) guidelines (S1 CONSORT Checklist) [12].

### Patients

Potentially eligible participants were identified by clinicians at routine primary care visits at the 6 sites of the St Michael’s Hospital Academic Family Health Team in Toronto, Canada; the Assiginack Family Health Team and the Manitoulin Central Family Health Team on Manitoulin Island, Ontario, Canada; and the Huron Shores Family Health Team in Blind River Ontario. Patients aged 18 years or older who self-reported medication nonadherence due to cost in the last 12 months were eligible for inclusion. Study personnel asked: “In the last 12 months, did you not fill a prescription or do anything to make a prescription last longer because of the cost?” [10,13]. To avoid contamination, family members living at the same address of participants already enrolled in the study were excluded. We prevented people from joining a trial site in order to participate in the study by excluding those who joined within the 6 months. All enrolled participants provided written informed consent.

### Trial procedures

Allocation concealment was achieved using the REDCap online application [14]. Randomization was stratified by center and blocked using randomly permuted blocks of 2 and 4 [10]. Participants and care providers were aware of allocation group in this open trial. Outcomes were assessed in a blinded fashion. Regardless of the group of allocation, participants had their usual access to healthcare services that are generally publicly funded without any co-payments.

The intervention was free access to 128 essential medicines including antibiotics, analgesics, antipsychotics, antiretrovirals, glucose-lowering medicines, and antihypertensives (Text B in S1 Text). WHO’s 2013 model essential medicines list was adapted by a group of clinician-scientists based on local prescribing volumes, prescribing guidance, peer suggestions, and patient input [15,16]. A central community pharmacy was set up for the trial, and medicines were distributed by mail, except for time-sensitive medicines including anti-infectives, analgesics, diuretics, bronchodilators, antihypertensives, and antipsychotics that were immediately dispensed by prescribers at the point of care. Medicines that were not on the list, including controlled substances, could be accessed as usual.

Participants allocated to the control arm had their usual access to medicines at community pharmacies that could involve out-of-pocket payments for medicines, public insurance (with co-payments and deductibles), and private insurance (with co-payments and deductibles). Out-of-pocket medicine costs were estimated at CAN$452 per household per year in Canada in 2017, and the medicines forgone cost less than CAN$200 for 87% of those who report cost-related nonadherence and represented a wide range of therapeutic categories including treatments for mental health conditions (21%), pain (16%), cardiovascular disease (16%), and infectious diseases (15%) [17,18].

### Outcomes

The prespecified primary outcome was being adherent to all medicines that were appropriately prescribed at 24 months [10]. A participant was considered nonadherent to a prescription if either a review of primary care prescribing records or patient reports of the number of doses missed during the last week as reported by telephone interview or email survey between 21 and 24 months indicated that less than 80% of prescribed doses were taken. Chart abstraction was done by one adjudicator and verified by a second. We determined whether each prescription was potentially inappropriate using established criteria (e.g., warfarin prescribed with nonsteroidal anti-inflammatory) based only on the prescribed medicines (and not clinical characteristics such as renal function) (Text C in S1 Text) [19].

The 2 constituents of the primary outcome—adherence and appropriateness—were prespecified secondary outcomes. Three surrogate health outcomes were assessed as prespecified secondary outcomes using primary care records in participants taking the corresponding treatment, and all were adjusted for baseline: hemoglobin A1c levels, blood pressure, and low-density lipoprotein (LDL) cholesterol levels. Investigations were ordered as part of usual care. To assess the prespecified secondary outcome of healthcare costs, we used health administrative data from Ontario’s single-payer health system to ascertain the following outcomes: costs of ambulatory visits with primary care physicians, costs of ambulatory visits with specialist physicians, other physician costs including laboratory testing, costs of emergency department visits, costs of hospitalizations, costs of publicly funded medications, and costs of home care. Details are provided in Table E in S1 Tables. After a 21- to 24-month follow-up period, we asked patients a total of 14 questions about the provider–patient relationship, the information provided about medicines, medicine dispensing and delivery, the ability to make ends meet (pay for necessities), perceived health improvement, adverse effects of medicines, and frequency of healthcare visits (see Table D in S1 Tables for a list of the exploratory outcomes).

Serious adverse events were ascertained through reviews of the primary care charts.

### Statistical analysis

As previously described [10], we estimated sample sizes assuming that between 40% and 60% of control group participants would be appropriately adherent to all medications [8,20,21]. The maximum sample size to detect a 10% absolute difference (80% power, type 1 error 5%) was 392 per group, and this was obtained when the control proportion appropriately adherent was between 44% and 46%. Using the intention to treat principle, appropriate adherence was compared using a chi-squared test. We report the unadjusted treatment effect, and the absolute risk difference with 95% confidence interval (CI) and a *p*-value of <0.05 was used to reject the null hypothesis of no difference. Poisson regression was used for the adherence and potentially inappropriate prescriptions, analysis of covariance was used for the surrogate health outcomes (hemoglobin A1c, blood pressure, and LDL cholesterol), and the Kruskal–Wallis test was used for healthcare costs. During the peer review process, we conducted an analysis of the primary outcome adjusting for age, sex, site, and income.

## Results

### Patients

**Fig 1:**
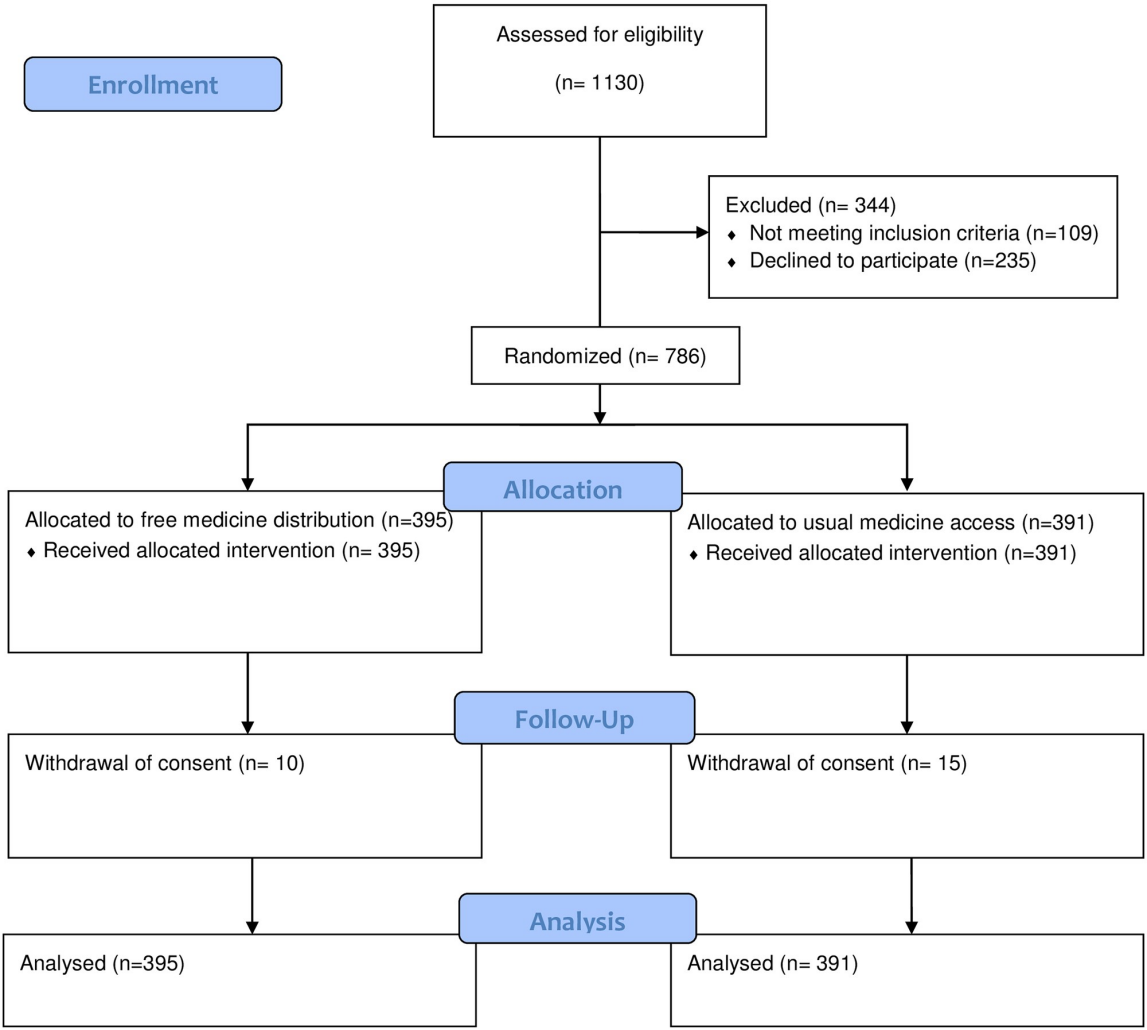
Participant flow diagram.

The trial population was recruited between June 1, 2016 and April 28, 2017. A total of 1,030 individuals identified as potentially eligible by clinicians were assessed for eligibility, and 786 were randomly allocated (Fig 1). For the 25 of 786 (3.2%) participants who withdrew consent, 10 of 395 (2.5%) in the free distribution arm, and 15 of 391 (3.8%) in the usual access arm, data collected prior to withdrawal were included in the analysis.

The trial population was mostly female (56%), younger than 65 years (83%), white based on self-identification (66%), and had a low income (below CAN$30,000, 48%), with wages as the primary source (56%) (Table 1). Table A in S1 Tables shows that participants at rural sites were more likely to be white and older than 65 years and Table B shows that the common medicines prescribed to participants in both groups at baseline included analgesics, diabetes treatments, proton pump inhibitors, hypertension treatments, and puffers for asthma and chronic obstructive pulmonary disease.

**Table 1:** Baseline participant characteristics by group.

	Free distribution number (%) (*n* = 395)	Usual access number (%) (*n* = 391)
Women	220 (55.7)	219 (56.0)
Age (mean, SD, median, IQR)	51.0 ± 14.2	50.4 ± 14.3
Age 65 years or older	71 (18.0)	64 (16.4)
Ethnicity		
White	256 (64.8)	260 (66.5)
Black	35 (8.9)	39 (10.0)
Southeast or East Asian (including Korean, Japanese, Filipino, and Chinese)	28 (7.1)	19 (4.9)
South Asian	25 (6.3)	24 (6.1)
Latin American	10 (2.5)	15 (3.8)
Indigenous	12 (3.0)	14 (3.6)
West Asian (including Arab)	6 (1.5)	5 (1.3)
Mixed or other	22 (5.6)	8 (2.0)
Declined to provide	1 (0.3)	7 (1.8)
Main income source		
Wages and salaries (including self-employed)	218 (55.2)	221 (56.5)
Pension	50 (12.7)	42 (10.7)
Social support (e.g., welfare or disability)	36 (9.1)	47 (12.0)
Unemployment insurance	15 (3.8)	9 (2.3)
Other	56 (14.2)	51 (13.0)
Declined to provide	|20 (5.1)	21 (5.4)
Household income		
CAN$30,000 or less	205 (51.9)	182 (46.5)
CAN$30,000 to CAN$70,000	92 (23.3)	99 (25.3)
CAN$70,000 or greater	21 (5.3)	22 (5.6)
Number of medicines prescribed at baseline	5.27 ± 3.60	5.62 ± 3.99
Urban site	269 (68.1)	267 (68.3)
Rural site	126 (31.9)	124 (31.7)
Prescribed a diabetes treatment	89 (22.5)	91 (23.3)
Prescribed an antihypertensive	122 (30.9)	114 (29.2)
Prescribed a statin	81 (20.5)	81 (20.7)

### Adherence

The effects of the intervention on medicine adherence and prescribing appropriateness are shown in Table 2. After 2 years, adherence to all appropriate prescribed medicines was 38.7% in the free distribution group and 28.6% in the usual access group, a relative increase of 35.3%. The adjusted difference between groups (10.1%; 95% CI 3.3% to 16.9%) was as large as our prespecified definition of an important difference (10%) and statistically significant (*p* = 0.004). The effect of the free distribution on adherence with adjustment for age, sex, site, and income level (odds ratio 1.56; 95% CI 1.15 to 2.12; *p* = 0.004) was similar to an unadjusted model (odds ratio 1.57; 95% CI 1.17 to 2.12; *p* = 0.003). Adherence was higher based on patient report compared with chart reviews (see Table C in S1 Tables).

**Table 2:** Primary and secondary outcomes results by group after 2 years.

	Free distribution (*n* = 395)	Usual access (*n* = 391)	Difference	*p*-value
*Primary outcome*				
Participants appropriately adherent to all medicines	153 (38.7%)	112 (28.6%)	10.1%; 95% CI 3.3% to 16.9%	0.004
*Secondary outcomes*				
Mean percentage of medicines adhered to by each participant*	74.5%	66.6%	7.9%; 95% CI 0.66% to 15.7%	0.037
Mean percentage of medicines potentially inappropriately prescribed to by each participant*	0.42%	1.43%	−1.00%; 95% CI −1.24% to −0.46%	0.005

*Differences estimated from rate ratios and estimated mean percentage in control group. Rate ratio for medicines adhered to was 1.12 (95% CI 1.01 to 1.24). Rate ratio for potentially inappropriate prescriptions was 0.31 (95% CI 0.13 to 0.67).

### Secondary outcomes

The clinical surrogate health outcomes in the subset of patients prescribed treatments for diabetes, treatments for hypertension, or statins are shown in Table 3. There was substantial missing data for all surrogate health outcomes because testing results were not available (36% for diabetes, 37% for blood pressure, and 52% for cholesterol), and missing data were less common in the free distribution group compared with the control group (28% versus 44% for diabetes, 31% versus 44% for blood pressure, and 46% versus 58% for cholesterol). There were no statistically significant differences in surrogate health outcomes based on the available data.

**Table 3:** Secondary surrogate health outcome results by group after 2 years.

	Free distribution	Usual access
Hemoglobin A1c (%)	(*n* = 64)	(*n* = 51)
Baseline	8.08 ± 1.68	8.15 ± 1.78
Follow-up	7.93 ± 1.39	7.68 ± 1.66
Difference	0.27; 95% CI −0.25 to 0.79 (*p* = 0.302)	
Blood pressure, systolic (mm Hg)	(*n* = 84)	(*n* = 64)
Baseline	137 ± 17	136 ± 17
Follow-up	134 ± 19	138 ± 19
Difference	−3.9; 95% CI −9.9 to 2.2 (*p* = 0.210)	
Blood pressure, diastolic (mm Hg)	(*n* = 84)	(*n* = 64)
Baseline	80 ± 12	81 ± 10
Follow-up	80 ± 11	80 ± 10
Difference	−0.01; 95% CI −3.1 to 3.1 (*p* = 0.993)	
LDL cholesterol, (mmol/L)	(*n* = 44)	(*n* = 34)
Baseline	2.2 ± 0.9	2.1 ± 1.1
Follow-up	2.0 ± 0.9	1.7 ± 0.8
Difference	0.26; 95% CI −0.08 to 0.60 (*p* = 0.130)	

LDL, low-density lipoprotein.

Healthcare costs were available for 747 (95%) participants who consented to the analysis of administrative data (382/395 or 97% in the free distribution group and 365/391 or 93% in the usual access group). Free distribution lowered total healthcare costs by a median of CAN$1,117 (95% CI CAN$445 to CAN$1,778, *p* = 0.006) or 38%. Total healthcare costs in the free distribution group (median CAN$1,782 [interquartile range, CAN$594 to CAN$5,854], mean CAN$9,112 ± CAN$21,904) and the usual access group (CAN$2,899, [interquartile range, CAN$901 to CAN$9,744], mean CAN$11,556 ± CAN$28,630) were low (<CAN$4,000) for most participants (461 or 62%) in both groups, and a relatively small proportion (93 or 12%) had high (>CAN$18,000) costs. Healthcare spending in all categories except home care costs were lower in the free distribution group, and hospitalizations accounted for the largest share of spending (see Table E in S1 Tables).

### Other outcomes

The effects of the intervention on the 14 exploratory patient-oriented outcomes are shown in Table D in S1 Tables. There were statistically significant effects favoring the intervention for 10 outcomes, differences that were not statistically significant with a trend favoring the intervention for 2 outcomes, and neutral effects that were not significantly different for 2 outcomes. Based on these patient reports, the 3 largest effects of the intervention were in the ability to “make ends meet” or afford basic necessities (57.8% absolute increase; 95% CI 50.5% to 65.1%), care quality (31.5% absolute increase; 95% CI 23.7% to 39.2%), and overall health (30.9% absolute increase; 95% CI 22.8% to 39.0%).

### Safety

In the free distribution group, 51 participants experienced a total of 88 serious adverse events, while in the usual access group, 68 participants experienced a total of 114 serious adverse events (*p* = 0.091 for number of participants experiencing at least 1 serious adverse event). There were 8 deaths in the free distribution group and 10 deaths in the control group (*p* = 0.795). There were 39 medication incidents in the intervention group, mostly related to medication delivery. Medication incidents were not monitored in the usual access group.

## Discussion

In this randomized trial involving 786 primary care patients in Ontario, Canada who reported cost-related nonadherence, free distribution of essential medicines improved medicine adherence after 2 years. Access to appropriately prescribed medicines improved without any increase in potentially inappropriate prescribing using a narrow definition.

Unlike other studies of providing free access to both care and medicines to uninsured individuals, our trial isolated the effects of free medicine distribution because participants in both groups had access to publicly funded healthcare services such as hospitalizations, physician visits, and diagnostic investigations. The results of this trial therefore provide information about the medium- to long-term effects of free medicine distribution to primary care patients who have trouble affording medicines, regardless of their income, insurance status, or whether they live in an urban or rural community.

We observed a larger improvement in adherence over a longer follow-up (10.1% over 2 years; number needed to treat of 10) compared with the 2 previous trials with shorter follow-up in patients who had a myocardial infarction: MI FREEE (5.4% absolute increase over median follow-up of 394 days) and ARTEMIS (3.3% absolute increase over 1 year follow-up) [8,9]. The population in our trial were primary care patients who reported cost-related nonadherence and a comprehensive set of medicines were provided as opposed to secondary myocardial infarction prevention treatments. An observational study found that public health insurance including medication insurance was associated with a reduction in missing medicine doses due to the cost (11.6% over 3 years of difference-in-difference study) [22]. Some of the few interventions that improve adherence, including combining multiple medicines for cardiovascular disease prevention into a single “polypill” and offering financial incentives to patients for taking medicines as directed, have effects comparable in magnitude to the effect observed in our trial [23–25].

While free distribution of medicines increased adherence substantially, the fact that most participants in the intervention group did not take all prescribed treatments as directed indicates that factors other than out-of-pocket cost are important contributors to nonadherence. Other studies have found that adherence is related to access to healthcare services, the clarity of information about medicines, and the patient–provider relationship [26]. In a quasi-experimental study, a value-based insurance design that provided free access to some chronic disease treatments improved adherence overall, but there was no benefit for those living in low-income neighborhoods [27].

The increase in reported ability to make ends meet, improved care, and patient-reported overall health were substantially larger than the increase in the primary outcome of adherence, suggesting that free medicine provision may have indirect effects even when it does not improve medicine adherence above the commonly used threshold of taking at least 80% of expected doses. Findings based on these exploratory outcomes should be interpreted carefully, and further study is needed. The Oregon Health Experiment similarly showed that random allocation to an invitation to apply for public insurance (Medicaid) reduced financial strain and improved self-reported health but did not substantially affect chronic disease management including diabetes control [28]. Even relatively small out-of-pocket medicine costs can reduce adherence based on observational studies [29,30].

Free medicine distribution reduced total healthcare costs that includes hospitalization costs over 2 years. This finding is consistent with a real health improvement, although there was no difference in the surrogate health outcomes based on the available data, and reduced healthcare utilization does not necessarily mean better health. The longer-term effects on healthcare utilization and costs might be different than those observed over 2 years; the effect could wane if adherence diminishes or the effect could grow as the benefits of improvements in adherence to long-term treatments such as antihypertensives accrue. The fact that improvement in adherence was maintained over 2 years means a long-term benefit is plausible. Considering only pharmaceutical spending, publicly funding essential medicines in Canada is projected to save CAN$4.3 billion annually due to lower drug prices with an increase in public drug spending of CAN$1.2 billion annually [31].

### Limitations

The group of allocation could not be kept from participants, and allocation to free distribution of medicines might have motivated participants to adhere, although the effects on healthcare costs is not easily explained by patient motivation. Medicine adherence was ascertained using patient report and reviews of prescriptions in medical records, 2 common approaches that can, respectively, over- and underestimate adherence, but the effect of the intervention was similar regardless of how the primary outcome was defined. Similar to other studies of interventions to improve adherence to proven medicines [24], this trial was not powered to detect differences in clinical outcomes such as death, since taking proven treatments for conditions such as hypertension and HIV is known to reduce mortality. Medicines were not only provided for free, but they were also provided conveniently to participants in the intervention group, and this 2-arm trial was not designed to assess the effect of each component of the intervention. A substantial number of participants receiving chronic disease treatments had missing surrogate health outcome data as we did not require measurement of these secondary outcomes because doing so may have affected the primary outcome of adherence; the effect of the intervention on surrogate clinical outcomes could have truly waned or even changed direction after 1 year [10], or the large amount of imbalanced missing data could have obscured an effect that was durable after 2 years. Death represented a competing risk for outcome ascertainment including for surrogate health outcomes, although the rate of death was relatively low (2%) during the 2-year trial. We assessed only routinely collected surrogate clinical outcomes that did not assess control of respiratory, rheumatologic, mental health, and other chronic conditions. Since the intervention involved free provision of only a short list of essential medicines, the results including the effect on prescribing appropriateness may not be applicable to longer lists of publicly funded medicines. We employed a narrow definition of potentially inappropriate prescribing. We did not assess baseline medicine adherence, medicine costs, or total health spending, although adjusting for other baseline characteristics did not change the results of this trial. The effects of this type of intervention may vary in different settings based on access to healthcare services, medicine costs, and other factors.

## Conclusions

While considering these limitations and the strengths of this trial, the findings can inform discussions about coverage for medicines. Global plans to move toward universal health coverage include improved access to essential medicines [7]. There is a proposal in Canada to include a list of essential medicines in the publicly funded healthcare system while reducing total pharmaceutical spending through increased purchasing power [31,32]. Prescription medicines are also included in “Medicare for All” proposals in the United States that are hotly contested [33]. The results of our study indicate that multiple potential effects of such changes should be carefully tracked.

## Supporting information

S1 Text Contributors, medicines included in intervention and criteria for identifying potentially inappropriate prescriptions. (DOCX)

S1 Tables Baseline participant characteristics by setting, prescribed medicines results, alternative definitions of primary outcome results, patient oriented outcomes results, health care costs results. (DOCX)

S1 CONSORT Checklist Checklist of information to include when reporting the results of a randomized controlled trial. (DOC)

## Abbreviations

ARTEMIS: Affordability and Real-world Antiplatelet Treatment Effectiveness After Myocardial Infarction Study
CI: confidence interval
CLEAN Meds: Carefully seLected and Easily Accessible at No Charge Medications
CONSORT: CONsolidated Standards of Reporting Trials
LDL: low-density lipoprotein
MI FREEE: Post-Myocardial Infarction Free Rx Event and Economic Evaluation
