# Supplementary material for: Adherence at 2 years with distribution of essential medicines at no charge: The CLEAN Meds randomized clinical trial
Source: PLoS Med. 2021 May 21;18(5):e1003590. doi: 10.1371/journal.pmed.1003590 (PMC8139488; doi:10.1371/journal.pmed.1003590)
Supplement: S1 Text — (DOCX) [file pmed.1003590.s001.docx]

# **Text A**

# Contributors and members of the study team

Study pharmacist: Norman Umali

Study team members: Itunu Adekoya, Haroon Ahmed, Darshanand Maraj, Anjli Bali, Taehoon Lee, Efosa Oronsaye, Yathavan Rajakulasingam, Liane Steiner, Sapna Wadhawan, Hannah Woods, Hannah Yaphe

Data Safety Monitoring Board members: Dr Dee Mangin (chair), Dr William Ghali, Dr Dean Fergusson, and Dr Andrew Day. Administrative support of the Data Safety Monitoring Board: Patricia Habran Dietrich, Jodi Peters, Karen Green.

Community Guidance Panel members: Diane Charter, Marleane Davidson, Margaret Golding, Sureya Ibrahim, Linda Stella, and others who did not provide consent to be named.

Clinicians: Nada Abdel-Malek, Zoe von Aesch, Mouafak Al Hadi, Kelly Anderson, Gordon Arbess, Chris Barnes, Peter Barreca, Seema Bhandarkar, Gary Bloch, Tali Bogler, Ashna Bowry, Donnavan Boyd, Marc Bradford, Anne Browne, Paul Das, MaryBeth Derocher, Katie Dorman, Kathleen Doukas, Esther Ernst, Allison Farber, Hannah Feiner, Amy Freedman, Kari Fulton, Chantal Gaudreau, Abbas Ghavam-Rassoul, Rajesh Girdhari, Richard Glazier, Irv Gora, Kimberley Gordon, Laurie Green, Samantha Green, Charlie Guiang, Curtis Handford, Maryna Harelnikiva, Candice Holmes, Sue Hranilovic, Karl Iglar, Gwen Jansz, Emma Jeavons, Nick Jeeves, Frances Kilbertus, Flo Kim, Tara Kiran, Holly Knowles, Bruce Kwok, Sheila Lakhoo, Margarita Lam-Antoniades, Renata Leong, Fok-Han Leung, Aisha Lofters, Jennifer McCabe, Lora McDougall, Joanne Mellan, Sharon Mintz, Matthew Naccarato, Maya Nader, Kevin O'Connor, James Owen, Judith Peranson, Andrew Pinto, Cristina Pop, Adam Pyle, Julia Rackal, Noor Ramji, Nasreen Ramji, Danyaal Raza, Maurianne Reade, Jane Ridley, Jean Robison, Katherine Rouleau, Caroline Ruderman, Vanna Schiralli, Lee Schofield, Mary Shamas, Susan Shepherd, Rami Shoucri, Lenka Snajdrova, Andrew Stadnyk, Ann Stewart, Bill Sullivan, Karen Swirsky, Joshua Tepper, Suzanne Turner, Barbara Vari, Priya Vasa, Karim Vellani, Tao Wang, William Watson, Thea Weisdorf, Karen Weyman, Sheila Wijayasinghe, Jean Wilson, and Patricia Windrim

# **Text B**

# Essential Medicines Provided to Patients With Free Distribution

- acetaminophen
- acetylsalicylic acid
- adalimumab
- alendronate
- allopurinol
- amiodarone
- amlodipine
- amoxicillin
- amoxicillin/clavulanic acid
- atomoxetine
- atorvastatin
- azathioprine
- azithromycin
- baclofen
- beclomethasone
- benzoyl peroxide
- benztropine
- betamethasone
- bevacizumab
- bisoprolol
- budesonide
- candesartan
- carbamazepine
- cephalexin
- cetirizine
- chlorthalidone
- ciprofloxacin
- clarithromycin
- clindamycin
- clopidogrel
- clotrimazole
- cloxacillin
- clozapine
- conjugated estrogens
- copper (intrauterine device)
- dabigatran
- dexamethasone
- diltiazem
- dolutegravir
- domperidone
- donepezil
- doxycycline
- efavirenz
- eletriptan
- emtricitabine
- epinephrine
- estradiol
- ethinyl estradiol/levonorgestrel
- ferrous fumarate
- finasteride
- fluconazole
- fluoxetine
- fluticasone
- folic acid
- furosemide
- fusidic acid
- gabapentin
- gliclazide
- haloperidol
- hydrocortisone
- hydroxychloroquine
- ibuprofen
- insulin, long acting
- insulin, short acting
- ipratropium
- labetalol
- lamivudine
- latanoprost
- levodopa/carbidopa
- levofloxacin
- levonorgestrel
- levothyroxine
- lithium
- loperamide
- medroxyprogesterone
- metformin
- methimazole
- methotrexate
- methylprednisolone
- metoclopramide
- metronidazole
- mupirocin
- naltrexone
- naproxen
- nicotine replacement therapy
- nitrofurantoin
- nitroglycerin
- nortriptyline
- nystatin
- olopatadine
- pantoprazole
- permethrin
- phenytoin
- pilocarpine
- polyethylene glycol 3350
- polymyxin B
- potassium
- pravastatin
- prednisone
- propylthiouracil
- ramipril
- ranitidine
- risperidone
- rivaroxaban
- salbutamol
- salicylic acid
- salmeterol
- senna
- sertraline
- spironolactone
- sulfamethoxazole/trimethoprim
- sulfasalazine
- tadalafil
- tamsulosin
- tenofovir disoproxil fumarate
- thiamine
- tiotropium
- tranexamic acid
- tretinoin
- trimethoprim
- urea
- vaginal ring eluting etonogestrel and ethinyl estradiol
- valacyclovir
- valproic acid
- varenicline
- vitamin B12
- vitamin D
- warfarin

**Reference**

Taglione MS, Ahmad H, Slater M, Aliarzadeh B, Glazier RH, Laupacis A, Persaud N. Development of a preliminary essential medicines list for Canada. CMAJ Open. 2017 Feb 16;5(1):E137-E143.

# **Text C**

# Criteria for Potentially Inappropriate Prescriptions

The following criteria was applied to medicine lists for each patient.

- beta-blocker and cardioselective calcium channel blocker
- warfarin and non-steroidal anti-inflammatory
- direct-acting anticoagulant and non-steroidal anti-inflammatory
- warfarin and acetylsalicylic acid
- nitrate and phosphodiesterase inhibitor
- acetylsalicylic acid at doses greater than 160 mg per day
- glyburide at any dose (alternatives exist)
- duplicate class prescription (e.g. two thiazide diuretics simultaneously or two calcium channel blockers simultaneously)

**Reference**

O'Mahony D, O'Sullivan D, Byrne S, O'Connor MN, Ryan C, Gallagher P. STOPP/START criteria for potentially inappropriate prescribing in older people: version 2. *Age Ageing*. 2014;44(2):213-8.
