# Supplementary material for: Adherence at 2 years with distribution of essential medicines at no charge: The CLEAN Meds randomized clinical trial
Source: PLoS Med. 2021 May 21;18(5):e1003590. doi: 10.1371/journal.pmed.1003590 (PMC8139488; doi:10.1371/journal.pmed.1003590)
Supplement: S1 Tables — (DOCX) [file pmed.1003590.s002.docx]

Table A. Baseline participant characteristics by setting

|  | Urban  Number (%)  (n = 536) | Rural  Number (%)  (n = 250) |
| --- | --- | --- |
| Women | 302 (56.3) | 137 (54.8) |
| Age (mean, SD, median, IQR) | 48.8 ± 14.1 | 54.9 ± 13.8 |
| Age 65 years or older | 76 (14.2) | 59 (23.6) |
| Ethnicity |  |  |
| White | 277 (51.7) | 239 (95.6) |
| Black | 74 (13.8) | 0 (0.0) |
| Southeast or East Asian (incl |  |  |
| Korean, Japanese, Filipino, Chinese) | 47 (8.8) | 0 (0.0) |
| South Asian | 47 (8.8) | 2 (0.8) |
| Latin American | 25 (4.7) | 0 (0.0) |
| Indigenous | 19 (3.5) | 7 (2.8) |
| West Asian (including Arab) | 11 (2.1) | 0 (0.0) |
| Mixed or other | 29 (5.4) | 1 (0.4) |
| Declined to provide | 7 (1.3) | 1 (0.4) |
| Main Income source |  |  |
| Wages and salaries (including self- | 295 (55.0) | 144 (57.6) |
| employed) |  |  |
| Pension | 47 (8.8) | 45 (18.0) |
| Social support (e.g. welfare or | 67 (12.5) | 16 (6.4) |
| disability) |  |  |
| Unemployment insurance | 13 (2.4) | 11 (4.4) |
| Other | 88 (16.4) | 19 (7.6) |
| Declined to provide | 26 (4.9) | 15 (6.0) |
| Household income |  |  |
| $30 000 CAD or less | 301 (56.2) | 86 (34.4) |
| $30 000 to 70 000 | 117 (21.8) | 74 (29.6) |
| $70 000 or greater | 33 (6.2) | 10 (4.0) |
| Number of medicines prescribed at | 5.37 ± 3.72 | 5.62 ± 3.99 |
| baseline |  |  |

# Table B. Prescribed medicines by Anatomical Therapeutic Chemical Classification System main groups

| Anatomical Therapeutic Chemical main groups (examples of medicines commonly prescribed) | Free distribution, number of prescriptions (% of prescriptions) | Usual access  prescriptions (% of prescriptions) |
| --- | --- | --- |
| Nervous system (gabapentin, sertraline, venlafaxine, acetaminophen) | 424 (20%) | 450 (20%) |
| Alimentary tract and metabolism (metformin, pantoprazole, rabeprazole, insulin) | 381 (18%) | 403 (18%) |
| Cardiovascular system (atorvastatin, ramipril, rosuvastatin, amlodipine, hydrochlorathiazide) | 326 (16%) | 366 (17%) |
| Respiratory system (salbutamol, fluticasone, tiotropium) | 274 (13%) | 264 (12%) |
| Dermatoligicals (hydrocortisone, betamethasone) | 161 (8%) | 159 (7%) |
| Blood and blood forming organs (acetylsalicylic acid, ferrous fumarate) | 125 (6%) | 140 (6%) |
| Musculo-skeletal system (naproxen, ibuprofen) | 117 (6%) | 128 (6%) |
| Genito urinary system and sex hormones (estradiol) | 116 (6%) | 124 (5%) |
| Antiinfectives for systemic use (amoxicillin) | 86 (4%) | 88 (4%) |
| Systemic hormonal preparations (levothyroxine) | 40 (2%) | 37 (2 %) |
| Other | 21 (1 %) | 24 (1 %) |

# Table C. Alternative Definitions of Primary Outcome Results Table

|  | Free distribution (N=395) | Usual access (N=391) | Difference | *P* value |
| --- | --- | --- | --- | --- |
| Participants adherent to all appropriately prescribed medicines (exclude any inappropriate prescriptions) | 156/384 (40.6 %) | 119/374 (31.8 %) | 8.8 %; 95 % CI 1.7 % to 15.9 % | 0.014 |
| Participants appropriately adherent to all medicines, excluding those with no regular medicines (those taking only as needed medicines) | 153/295 (51.9 %) | 112/243 (43.6 %) | 8.3 %; 95 % CI -0.004 % to 17.0 % | 0.063 |
| Participants adherent to all medicines, excluding those who withdrew | 153/384 (39.8 %) | 112/374 (29.9 %) | 9.9 %; 95 % CI 2.9 % to 16.9 % | 0.0054 |
| Participants appropriately adherent to all medicines, using only patient report data | 197/222 (88.7 %) | 132/156 (84.6 %) | 4.1 %; 95 % CI -3.5 % to 11.7 % | 0.31 |
| Participants appropriately adherent to all medicines, using only chart review data | 176/273 (64.5 %) | 127/240 (52.9 %) | 11.6 %; 95 % CI 2.7 % to 20.4 % | 0.010 |

# Table D. Patient Oriented Outcome Results Table

|  | Free distribution, N (%) | Usual access, N (%) | difference | *P* value |
| --- | --- | --- | --- | --- |
|  | (N=395) | (N=391) |  |  |
| Did you feel your care was better than before the study started? | 148/302 (49.0) | 43/245 (17.6) | 31.5 %; 95 % CI 23.7 % to 39.2 % | <0.0001 |
| Did you feel your questions or concerns were addressed by health care provider? | 290/301 (96.3) | 213/241 (88.4) | 8.0 %; 95 % CI 3.0 % to 12.9 % | 0.00068 |
| Was your interaction with specialists different from before the study started? | 52/292 (17.8) | 33/239 (13.8) | 0.04 %; 95 % CI -3.6 % to 10.6 % | 0.26 |
| Did the information about medications given by the pharmacist and the doctor match? | 294/301 (97.7) | 224/246 (91.1) | 6.6 %; 95 % CI 2.3 % to 10.9 % | 0.0012 |
| Was it easy to obtain information about the medications? | 302/303 (99.7) | 229/244 (93.9) | 5.8 %; 95 % CI 2.4 % to 9.3 % | 0.00017 |
| Did the information change the way you took medications? | 85/302 (28.1) | 54/245 (22.0) | 6.1 %; 95 % CI -1.5 % to 13.7 % | 0.12 |
| Did you have unanswered questions about your medications? | 9/305 (3.0) | 16/246 (6.5) | -3.5 %; 95 % CI -7.5 % to 0.4 % | 0.074 |
| Was the medication in good condition when you received it? | 304/304 (100) | 236/245 (96.3) | 3.7 %; 95 % CI 0.9 % to 6.4 % | 0.0024 |
| Did you receive new medications quickly? | 275/297 (92.6) | 207/236 (87.7) | 4.9 %; 95 % CI -0.64 % to 10.4 % | 0.079 |
| Did you receive the medication before the previous prescription ran out? | 249/284 (87.7) | 152/235 (64.7) | 23.0 %; 95 % CI 15.4 % to 30.6 % | <0.0001 |
| Was it easier to make ends meet at the end of the month? | 261/302 (86.4) | 67/234 (28.6) | 57.8 %; 95 % CI 50.5 % to 65.1 % | <0.0001 |
| Do you feel your health improved? | 240/296 (81.1) | 122/243 (50.2) | 30.9 %; 95 % CI 22.8 % to 39.0 % | <0.0001 |
| Did you have any medication side effects? | 73/303 (24.1) | 86/245 (35.1) | -11.0 %; 95 % CI -19.1 % to -2.3 % | 0.0064 |
| Did you have fewer healthcare visits? | 171/300 (57.0) | 95/246 (38.6) | 18.4 %; 95 % CI 9.7 % to 27.0 % | <0.0001 |

Table E. Health care costs by group over two years

|  | Free distribution, $ | | Usual access, $ | |
| --- | --- | --- | --- | --- |
|  | median (IQR) | mean ± S.D. | median (IQR) | mean ± S.D. |
| Primary care visits | 67 (0-184) | 159 ± 358 | 67 (10-201) | 281 ± 1,017 |
| Consultant visits | 237 (58-647) | 538 ± 948 | 282 (73-798) | 659 ± 1,121 |
| Emergency department | 156 (0-641) | 625 ± 1,409 | 209 (0-835) | 674 ± 1,460 |
| Hospitalization | 0 (0-877) | 3,994 ± 14,151 | 0 (0-3,099) | 5,661 ± 21,828 |
| Publicly funded medicines | 8 (0-901) | 2,284 ± 9,983 | 68 (0-1568) | 2,721 ± 8,885 |
| Homecare | 0 (0-0) | 811 ± 4,201 | 0 (0-0) | 627 ± 3,536 |
| Other (outpatient investigations) | 363 (139-932) | 702 ± 1,028 | 453 (145-916) | 933 ± 2,765 |
| Total | 1,782 (594-5854) | 9,112 ± 21,904 | 2,899 (901-9744) | 11,556 ± 28,631 |
